# The association between breast fibrosis, cosmetic outcomes, and long-term health-related quality of life after breast-conserving therapy: a multicenter cross-sectional observational cohort study

**DOI:** 10.1016/j.breast.2025.104541

**Published:** 2025-07-14

**Authors:** M.C.A.W. Notenboom, T.M.A.L. Klem, C.M.E. Contant, S.P. Ribbe, M. Franckena, J.J. Penninkhof, L.B. Koppert, P.W. Plaisier, M.A.M. Mureau, E.D. van Werkhoven, F.J.C. van der Veen, M. de Kraker, R.A. Nout, M.B.E. Menke-Pluijmers, F.E. Froklage

**Affiliations:** a Department of Radiotherapy, Erasmus MC Cancer Institute, University Medical Centre, Rotterdam, the Netherlands; b Department of Surgery, Albert Schweitzer Hospital, Dordrecht, the Netherlands; c Department of Surgery, Franciscus Gasthuis & Vlietland, Rotterdam, the Netherlands; d Department of Surgery, Breast Center South Holland South Maasstad Hospital, Rotterdam, the Netherlands; e Department of Surgery, Erasmus MC Cancer Institute, University Medical Centre, Rotterdam, the Netherlands; f Department of Plastic and Reconstructive Surgery, Erasmus MC Cancer Institute, University Medical Centre, Rotterdam, the Netherlands; g Department of Hematology, Erasmus MC Cancer Institute, University Medical Centre, Rotterdam, the Netherlands; h Department of Plastic and Reconstructive Surgery, Albert Schweitzer Hospital, Dordrecht, the Netherlands; i Department of Plastic and Reconstructive Surgery, Franciscus Gasthuis & Vlietland, Rotterdam, the Netherlands

**Keywords:** Breast cancer, Breast-conserving therapy, Breast fibrosis, Cosmetic outcomes, Health-related quality of life

## Abstract

**Background:**

Breast fibrosis is a well-known late side-effect of breast-conserving therapy (BCT) and may lead to breast retraction, asymmetry, and pain. Since life expectancy of breast cancer patients has significantly improved in the past decades, cosmetic outcomes and health-related quality of life (HRQoL) have gained importance. This study aimed to investigate the association between breast fibrosis, cosmetic outcomes, and various HRQoL domains.

**Methods:**

In this multicenter, cross-sectional, observational cohort (STARLINGS study), breast fibrosis was assessed (CTCAE version 5), breast photos were analyzed with BCCT.core software, and participants completed BREAST-Q, EORTC QLQ-BR23/C30, and 9-item cosmetic questionnaire. Associations between breast fibrosis and HRQoL and between cosmetic outcomes and HRQoL, were analyzed using multivariable linear regression, both unadjusted and adjusted for age, smoking and body mass index.

**Results:**

A total of 775 patients treated between 2016 and 2020 were included, with median follow-up of 4 years. Compared to patients with moderate/severe breast fibrosis, patients with none/mild breast fibrosis reported better HRQoL on all domains, except Sexual Functioning, Sexual Enjoyment, and Physical and Social Functioning. Patients with excellent/good cosmetic outcomes reported better HRQoL than patients with fair/poor cosmetic outcomes on five out of 18 HRQoL domains, but not on any of the Symptoms domains.

**Conclusion:**

Our results indicate that breast fibrosis and unfavorable cosmetic outcomes are negatively associated with various HRQoL domains. Additionally, breast fibrosis is associated with locoregional symptoms and fatigue, whereas unfavorable cosmetic outcomes are not. This large multicenter study corroborates the interrelated nature of breast fibrosis, cosmetic outcomes, and HRQoL (ID: NCT05263362).

## Highlights

• Breast fibrosis and unfavorable cosmetic outcomes lead to lower scores in HRQoL domains.
• Breast fibrosis after breast-conserving therapy leads to local symptoms and fatigue.
• This association was not found for unfavorable cosmetic outcomes.
• Breast fibrosis and cosmetic outcomes are linked, but this was not a one-to-one association.

## 1 Introduction

Breast cancer is the most frequently diagnosed cancer among females in the world [1]. In the Netherlands, one in seven women will be diagnosed with breast cancer at some point during life [2]. The majority of breast cancer patients (60–70 %) can be treated with breast-conserving therapy (BCT) instead of mastectomy [[3], [4], [5]]. BCT consists of breast-conserving surgery (BCS) combined with a sentinel node procedure or axillary lymph node dissection, followed by radiotherapy with or without boost and systemic therapy if indicated. Life expectancy of breast cancer patients has significantly increased due to improved multimodality treatment in the past decades [3,4,6]. Oncoplastic surgical techniques for BCT have been developed and radiotherapy techniques have been improved and adopted in daily practice [7].

Unfortunately, breast fibrosis is a well-known late side effect of BCT, which occurs in a substantial subset of patients (10–30 %) with severe breast fibrosis in 2–5 % of patients [[8], [9], [10]]. Breast fibrosis may lead to breast retraction, breast asymmetry, and pain. Consequently, this can negatively influence cosmesis and health-related quality of life (HRQoL) [10,11]. Both clinicians and patients are eager to avoid these late effects. Recently, more awareness has risen among clinicians on the importance of patient-reported HRQoL after treatment [12,13]. However, limited long-term data is available on breast fibrosis, cosmetic outcomes, and patient-reported HRQoL in large cohorts of patients treated with contemporary oncoplastic surgery and radiotherapy techniques.

Therefore, we set up a multicenter observational cohort study, aiming to investigate the association between breast fibrosis and HRQoL, cosmetic outcomes and HRQoL, and breast fibrosis and cosmetic outcomes. We hypothesized that breast fibrosis negatively affects HRQoL and cosmetic outcomes, and that unfavorable cosmetic outcomes have a negative impact on HRQoL as well. These side effects can lead to lasting physical and emotional burdens for patients, making it essential to recognize their impact. Gaining insight into these effects is crucial for optimizing patient-tailored treatment and supporting meaningful shared decision-making.

## 2 Methods

### 2.1 Study design and patient population

The STARLINGS study (ClinicalTrials.gov ID: NCT05263362), is a multicenter cross-sectional observational cohort study with four participating Dutch hospitals, including three large teaching hospitals and one university hospital. Inclusion criteria were: female sex, age ≥18 years, treatment with BCT at one of the participating hospitals for non-metastatic, histologically proven invasive breast cancer (pT1-3N0-2aM0) or ductal carcinoma in situ (DCIS). BCS was performed between January 1st, 2016 and December 31st, 2020. Subsequently, all included patients received adjuvant whole breast radiotherapy at the university hospital as part of their BCT. The radiotherapeutic hypofractionation schedules (with or without boost) according to the Canadian and START B trial (i.e. 40.05 Gy in 15 fractions or 42.56 Gy in 16 fractions), and their corresponding boost schedule as widely implemented in the Netherlands (Supplementary Table 1), were applied in this study [14,15]. Exclusion criteria were: conventionally fractionated schemes (50 Gy in 25 fractions), any breast surgery of the ipsilateral breast after BCT, (pre-planned symmetrizing contralateral surgery was allowed), re-irradiation of the ipsilateral breast after BCT, proton therapy, progression of disease since BCT (and additional treatment) at time of possible inclusion, partial breast irradiation, pregnancy and/or breast feeding at time of possible inclusion. Written informed consent was obtained from all participating patients. This study was approved by the medical ethical committees of all participating hospitals (MEC-2021-0829).

### 2.2 Data collection

**Fig. 1:**
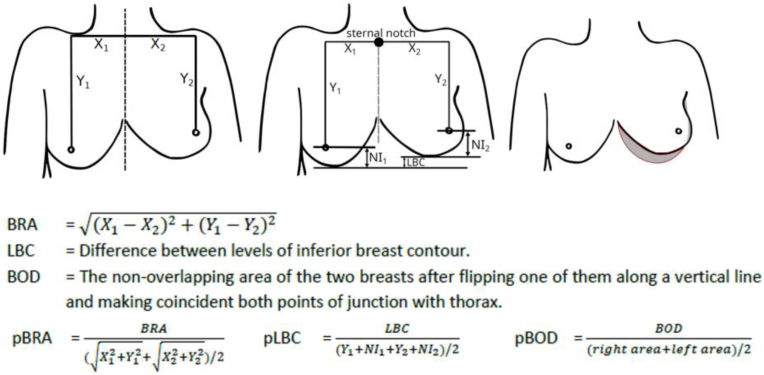
Schematic illustration of the cosmetic measurement and corresponding formulas used in BCCT.core software from Cardoso research group. From left to right: Breast Retraction Assessment (BRA), Lower Breast Contour (LBC), Breast Overlap Difference (BOD). The treated breast is compared to non-treated breast.

Eligible patients were identified from the electronic patient system and invited to participate in a study-information letter. Patients who did not respond were phoned (maximum of three attempts) to answer any questions and to ask to participate. After obtaining informed consent, participants made one study visit to their hospital. During this study visit, the maximum severity of fibrosis present in the breast was scored through physical examination using the Common Terminology Criteria for Adverse Events (CTCAE version 5) grading system. This was carried out by one trained physician researcher (M.C.A.W.N.), who also took standardized photographs of the breasts with patients’ arms along sides the body for frontal view (with a Nikon 300d camera). Beforehand, two skin marks were placed, one at the sternal notch and one 25 cm under the sternal notch, for the purpose of photograph evaluation with BCCT.core software [16]. BCCT.core evaluation was performed by two members of the research team (namely S.P.R. and all photos were checked by M.C.A.W.N.). The BCCT.core overall cosmetic evaluation was included in the analyses. Additionally, the three dimensionless asymmetry features known to be the most relevant variables, that represent various aspects of breast fibrosis, were used for analyses: Breast Retraction Assessment (BRA), Lower Breast Contour (LBC) and Breast Overlap Difference (BOD) (Fig. 1) [16,17].

Furthermore, smoking status and use of medication at time of BCT, bra size, and menopausal status at time of surgery were registered. All other information on patient and tumor characteristics, diagnosis, and treatment was retrospectively obtained through electronic medical records. After the study visit, patients once filled out four online HRQoL questionnaires at home, namely the BREAST-Q for BCT, EORTC QLQ-BR23/C30, and the 9-item cosmetic patient questionnaire [[18], [19], [20], [21], [22], [23], [24]]. Patients who were not able to fill out the questionnaires online, were provided with a paper version. For this study, five domains of the BREAST-Q were used, i.e. Physical Well-being, Sexual Well-being, Psychosocial Well-being, Satisfaction with Breasts and Adverse Effects of Radiation. The following six EORTC QLQ-BR23 domains were used: Body Image, Sexual Functioning, Sexual Enjoyment, Future Perspectives, Breast Symptoms, Arm Symptoms and for EORTC QLQ-BR23 seven domains: Global Health status, Physical Functioning, Role Functioning, Emotional Functioning, Social Functioning, Fatigue and Pain.

### 2.3 Statistical analysis

Breast fibrosis was dichotomized into ‘none/mild breast fibrosis’ and ‘moderate/severe breast fibrosis’ (according to CTCAE version 5 grade 0–1 and grade 2–3, respectively). For cosmetic outcomes evaluated by BCCT.core, the cohort was dichotomized based on the overall BCCT evaluation into ‘excellent/good’ and ‘fair/poor’ cosmetic outcomes. The relative values of the three dimensionless asymmetry features of BCCT.core software i.e. BRA, LBC and BOD, were considered continuous variables. Lower values of BRA, LBC and BOD represent less asymmetry, which usually correlates with better cosmetic outcomes. For the analyses of cosmetic outcomes (consisting of overall BCCT.core evaluation and relative BRA, LBC and BOD values), only unilaterally treated patients were included, because BCCT.core software has been developed for objective assessment of cosmetic breast outcomes by comparing the treated breast with the non-treated breast. Patients with a planned symmetrizing reduction mammoplasty were included in the cosmetic analyses.

Raw HRQoL scores of BREAST-Q, EORTC QLQ-BR23 and EORTC QLQ-C30 were transformed into scores that range from 0 to 100 and subsequently were considered as continuous variables. For BREAST-Q, EORTC QLQ-BR23 and EORTC QLQ-C30 domains, a higher score means better HRQoL or greater satisfaction, except for the Symptom domains, namely EORTC QLQ-BR23 domains Breast Symptoms and Arm Symptoms, and the EORTC QLQ-C30 domains Fatigue and Pain, where the opposite applies, i.e. a higher score means more symptoms. The 9-item cosmetic questionnaire consists of nine items each rated on a scale from 0 to 3. The average score was calculated and dichotomized where an average score of ≤1.5 was considered excellent/good and >1.5 was considered a fair/poor cosmetic outcome [18]. Incomplete questionnaire domains were excluded from the analyses of those specific domains. Agreement with BCCT.core was assessed using Cohen's kappa statistic. In literature no clear-cut threshold of Minimal Clinically Important Difference (MCID) for HRQoL questionnaires is reported. The MCID mostly ranges between 4 and 10 points on a HRQoL scoring ranging from 0 to 100 [[24], [25], [26]]. In this study, for BREAST-Q, EORTC QLQ-BR23/C30 a MCID of five points (delta Δ ≥ 5) was considered clinically relevant.

To assess the association between severity of breast fibrosis and the different HRQoL domains and between cosmetic outcomes and the different HRQoL domains, Mann-Whitney U tests were used for continuous HRQoL scores and Chi-squared tests for the dichotomized 9-item cosmetic questionnaire scores. To assess the association between breast fibrosis and HRQoL and the association between cosmetic outcomes and HRQoL further, multivariable linear regression was used, both unadjusted and adjusted for age, smoking and body mass index (BMI, kg/m^2^). To assess the association between breast fibrosis and cosmetic outcomes, logistic regression was used for the dichotomized BCCT.core evaluation (excellent/good and fair/poor cosmetic outcome) and linear regression for continuous BCCT.core cosmetic features (BRA, LBC, BOD). All statistical analyses were done in SPSS (version 25.0, Statistical Package for Social Sciences, Chicago, IL, USA). Two-sided p-values of <0.05 were considered statistically significant.

## 3 Results

**Fig. 2:**
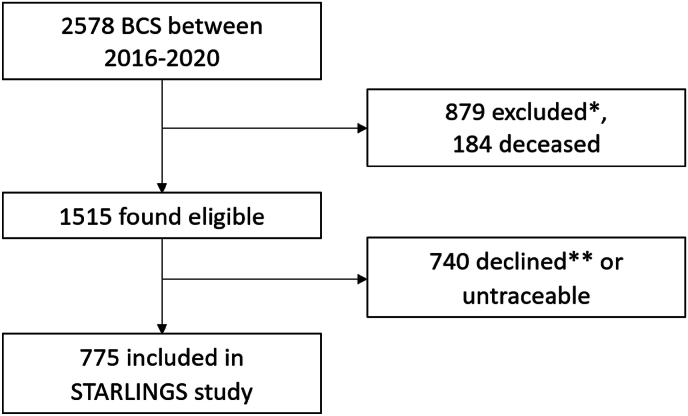
Flowchart of patient inclusion. ∗Did not meet inclusion criteria or met exclusion criteria. ∗∗Reasons for declining: busy schedule, too much stress or effort, no travel allowance, in a nursing home, dementia, does not want a picture taken because of religion, moved away, patient does not give reason. BSC = breast-conserving surgery.

A total of 2578 patients received BCS in one of the four participating hospitals between 2016 and 2020 with subsequent radiotherapy of the breast (Fig. 2). Of these 2578 patients, 879 did not meet the inclusion criteria or met exclusion criteria, and 184 were already deceased. Subsequently, 1515 eligible patients received the study information of whom 740 (48.8 % of total eligible) declined for various reasons or were untraceable. Finally, 775 patients gave informed consent and were included in the STARLINGS study.

### 3.1 Baseline characteristics

Median follow-up, calculated as time between surgery and date of inclusion, was 4 years (IQR: 3–5 years) (Table 1). The mean age at time of surgery was 57.5 (SD 10.2) years. Most women were post-menopausal (N = 505), with ACR density type B (N = 432) and main tumor location in the upper lateral quadrant (N = 396). All included patients received either simple oncoplastic reconstructive surgical techniques (reshaping of the breast without nipple displacement), volume displacement (with nipple displacement and breast reduction techniques) or volume replacement techniques (using tissue from outside of the breast) [27].

**Table 1:** Baseline characteristics.

	Total (N = 775)
Age at diagnosis (years)	
Mean age (SD)	57.5 (10.2)
Median (IQR)	58.0 (50.0–65.0)
Range (min-max)	27–81
Time between surgery and date of inclusion (years)	
Mean (SD)	4.5 (1.3)
Median (IQR)	4.0 (3.0–5.0)
Range (min-max)	2.0–8.0
Side breast in study	
Left	398 (51.4 %)
Right	377 (48.6 %)
BMI (kg/m^2^) at time of surgery	
Mean (SD)]	27.5 (5.1)
Median (IQR)	26.6 (23.8–30.1)
WHO performance status classification	
0	748 (96.5 %)
1	26 (3.4 %)
2	1 (0.1 %)
Smoking during BCT	
Yes	712 (91.9 %)
No	60 (7.7 %)
Unknown	3 (0.4 %)
Menopausal status at time of surgery	
Pre	179 (23.1 %)
Meno	69 (8.9 %)
Post	505 (65.2 %)
Unknown	22 (2.8 %)
Breast cup size	
A-C	392 (50.6 %)
D-F	296 (38.2 %)
G-J	23 (2.9 %)
Unknown	64 (8.3 %)
Breast circumference	
≤80 cm	246 (31.7 %)
81–90 cm	317 (40.9 %)
91–105 cm	133 (17.2 %)
Unknown	79 (10.2 %)
Diabetes Mellitus	
Type 1	2 (0.3 %)
Type 2	65 (8.4 %)
No	708 (91.4 %)
ACR Fibro glandular breast density	
Type A	88 (11.4 %)
Type B	432 (55.7 %)
Type C	206 (26.6 %)
Type D	36 (4.6 %)
Unknown	13 (1.7 %)
Clinical T stage	
T1	490 (63.2 %)
T2	183 (23.6 %)
T3	5 (0.6 %)
Tis	95 (12.3 %)
Unknown	2 (0.3 %)
Clinical N stage	
N0	500 (64.5 %)
N+	53 (6.8 %)
Unknown	222 (28.6 %)
Invasive breast cancer	
Yes	687 (88.6 %)
No	88 (11.4 %)
Carcinoma in situ present	
Yes	434 (56.0 %)
No	341 (44.0 %)
Maximum clinical invasive tumor/DCIS size(mm)	
Mean (SD)	17.2 (10.2)
Median (IQR)	15.0 (10.0–22.0)
≤10 mm	214 (27.6 %)
11–20 mm	331 (42.7 %)
21–50 mm	206 (26.6 %)
≥51 mm	6 (0.8 %)
Unknown	18 (2.3 %)
Histological type tumor	
NST/ductal	569 (73.4 %)
Lobular	85 (11.0 %)
Other	32 (4.1 %)
Unknown	89 (11.5 %)
Differentiation grade (Bloom Richardson)	
Grade 1	212 (27.4 %)
Grade 2	325 (41.9 %)
Grade 3	127 (16.4 %)
Unknown	111 (14.3 %)
Lymph angioinvasion	
Yes	60 (7.7 %)
No	508 (65.5 %)
Uncertain	36 (4.6 %)
Unknown	171 (22.1 %)
Hormone receptor/Her2 neu status^a^	
ER+	590 (76.1 %)
PR+	469 (60.5 %)
Her2Neu+	85 (11.0 %)
Localization main tumor^b^	
Upper lateral quadrant	396 (51.1 %)
Lower lateral quadrant	133 (17.2 %)
Upper medial quadrant	157 (20.3 %)
Lower medial quadrant	79 (10.2 %)
Central	74 (9.5 %)
Oncoplastic BCS technique	
Simple oncoplastic	702 (90.6 %)
Volume displacement	39 (5.0 %)
Volume replacement	34 (4.4 %)
Radiotherapy boost	
Yes	392 (50.6 %)
No	383 (49.4 %)
Systemic therapy^c^	
Yes	433 (55.9 %)
No	342 (44.1 %)

Numbers shown as N (%) unless stated otherwise. Some categories may not add up to 100 due to rounding. SD = Standard deviation; IQR = Interquartile range; BMI = Body mass index; WHO = World Health Organization; BCT = Breast-conserving therapy; ACR = American College of Radiology; DCIS = Ductal carcinoma in situ; NST = No special type; ER+ = Estrogen receptor positive; PR+ = Progesterone receptor positive; HER2Neu+ = human epidermal growth factor receptor 2 positive; BCS = breast-conserving surgery.

a Total percentage >100 % as some tumors had more than one positive receptor.

b Total percentage >100 % as some main tumors were in more than one quadrant.

c i.e. (neo)-adjuvant chemotherapy and/or targeted therapy and/or endocrine therapy.

Of all 775 photographs, 749 photographs were eligible for analysis of cosmetic outcomes, because 26 of the patients had a history of bilateral BCT who were therefore excluded. Patients with a planned symmetrizing reduction mammoplasty (N = 34) were eligible and included in the analyses. The response rates for the HRQoL questionnaires were high: 94.3 % for BREAST-Q and 9-item cosmetic questionnaire (N = 731), 93.3 % for EORTC QLQ-BR23 (N = 723), and 93.8 % for EORTC QLQ-C30 (N = 727). Incomplete questionnaire domains were excluded from the analyses of those specific domains. (Supplementary Table 2).

### 3.2 Association between severity of breast fibrosis and health-related quality of life

**Fig. 3:**
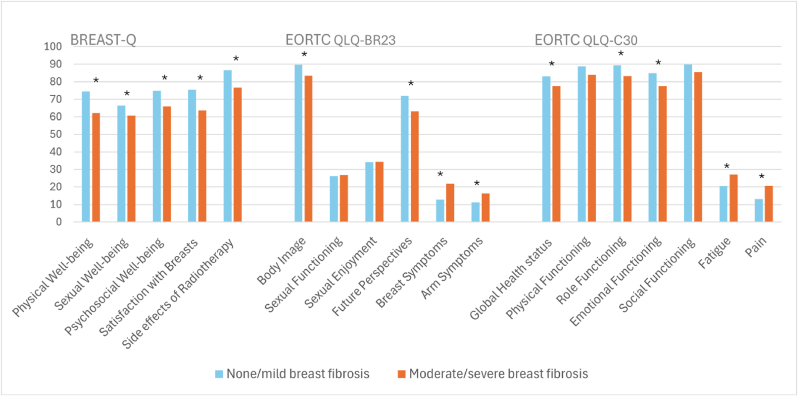
Mean health-related quality-of-life scores comparing patients with none/mild breast fibrosis and moderate/severe breast fibrosis ∗Difference clinically relevant and statistically significant. BREAST-Q Breast-Conserving Therapy module was used. EORTC-QLQ-BR23 = European Organization for Research and Treatment Quality of Life Questionnaire breast cancer module; EORTC-QLQ-C30 = European Organization for Research and Treatment Quality of Life Questionnaire Core 30.

Moderate/severe breast fibrosis was observed in 21 % of the cohort (N = 159), with no cases of grade 4 breast fibrosis. Compared to patients with moderate/severe breast fibrosis, patients with none/mild breast fibrosis reported a statistically significant as well as clinically relevant better HRQoL on all domains, except for the domains Sexual Functioning, Sexual Enjoyment, Physical Functioning and Social Functioning (Fig. 3 and Supplementary Table 3). The results of the univariable and adjusted multivariable regression analysis for breast fibrosis and HRQoL are shown in Table 2. Only Social Functioning became statistically not significant after adjusting for age, smoking, and BMI.

**Table 2:** Univariable and adjusted multivariable regression analysis with breast fibrosis as independent variable and health-related quality of life as dependent variable. Adjusted for age, smoking and BMI (kg/m^2^).

	Unadjusted		Adjusted	
	β (95% CI)	p-value	β (95% CI)	p-value
Breast-Q				
Physical Well-being	−12.34 (−16.10 to −8.58)	**<0.001**	−11.07 (−14.80 to −7.35)	**<0.001**
Sexual Well-being	−5.78 (−10.37 to −1.19)	**0.014**	−5.50 (−10.11 to −0.90)	**0.019**
Psychosocial Well-being	−8.93 (−13.78 to −4.08)	**<0.001**	−9.42 (−14.26 to −4.59)	**<0.001**
Satisfaction with Breasts	−11.94 (−15.27 to −8.61)	**<0.001**	−11.46 (−14.81 to −8.10)	**<0.001**
Side effects of Radiotherapy	−10.09 (−13.58 to −6.61)	**<0.001**	−8.61 (−12.04 to −5.18)	**<0.001**
EORTC QLQ-BR23				
Body Image	−6.36 (−9.79 to −2.93)	**<0.001**	−4.81 (−8.18 to −1.46)	**0.005**
Sexual Functioning	0.46 (−3.82 to 4.73)	0.834	−1.01 (−5.13 to −3.11)	0.630
Sexual Enjoyment	0.20 (−5.85 to −6.26)	0.066	−1.89 (−7.74 to −3.96)	0.526
Future Perspectives	−8.68 (−13.59 to −3.77)	**<0.001**	−6.72 (−11.56 to −1.89)	**0.007**
Breast Symptoms	9.15 (5.98–12.31)	**<0.001**	8.11 (4.99–11.24)	**<0.001**
Arm Symptoms	5.08 (1.53–8.63)	**0.005**	3.78 (0.33–7.25)	**<0.001**
EORTC QLQ-C30				
Global Health status	−5.59 (−8.66 to −2.52)	**<0.001**	−4.50 (−7.54 to −1.45)	**0.004**
Physical Functioning	−4.84 (−7.58 to −2.09)	**<0.001**	−4.64 (−7.34 to −1.94)	**<0.001**
Role Functioning	−5.93 (−9.57 to −2.28)	**0.001**	−5.55 (−9.23 to −1.88)	**0.003**
Emotional Functioning	−7.58 (−11.28 to −3.89)	**<0.001**	−6.56 (−10.25 to −2.86)	**<0.001**
Social Functioning	−4.40 (−7.97 to −0.83)	**0.016**	−3.31 (−6.87 to 0.25)	0.068
Fatigue	6.63 (2.62–10.64)	**0.001**	5.37 (1.38–9.36)	**0.008**
Pain	7.66 (3.77–11.56)	**<0.001**	6.73 (2.87–10.60)	**<0.001**
9-item cosmetic questionnaire	0.31 (0.22–0.40)	**<0.001**	0.30 (0.22–0.39)	**<0.001**

BREAST-Q Breast-Conserving Therapy module was used. EORTC-QLQ-BR23 = European Organization for Research and Treatment Quality of Life Questionnaire breast cancer module; EORTC-QLQ-C30 = European Organization for Research and Treatment Quality of Life Questionnaire Core 30; BMI = Body mass index; CI = Confidence Interval.

p-value statistically significant p <0.05 (stated in bold).

### 3.3 Association between cosmetic outcomes and health-related quality of life

**Fig. 4:**
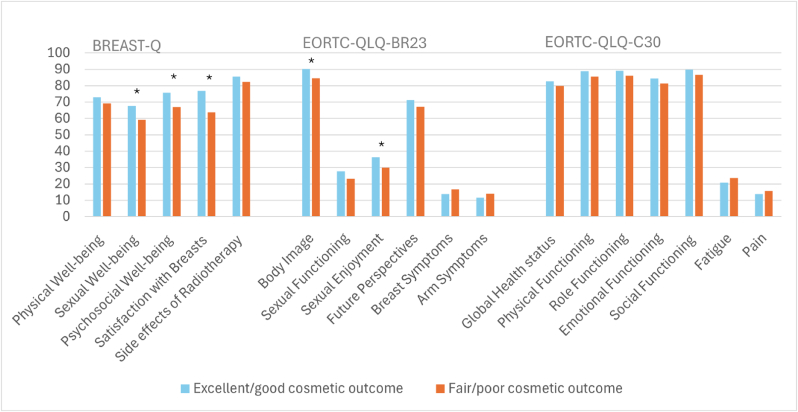
Mean health-related quality-of-life scores comparing patients with excellent/good cosmetic outcome and fair/poor cosmetic outcome. ∗Difference clinically relevant and statistically significant. BREAST-Q Breast-Conserving Therapy module was used. EORTC-QLQ-BR23 = European Organization for Research and Treatment Quality of Life Questionnaire breast cancer module; EORTC-QLQ-C30 = European Organization for Research and Treatment Quality of Life Questionnaire Core 30.

The association between cosmetic outcomes, in terms of dichotomized BCCT.core overall evaluation, and the different HRQoL domains is shown in Fig. 4 and Supplementary Table 4. A fair/poor cosmetic outcome was observed in 28 % (N = 213). Patients with excellent/good cosmetic outcome reported a statistically significant as well as clinically relevant better HRQoL than patients with fair/poor cosmetic outcome on five out of 18 HRQoL domains (i.e., Sexual Well-being, Psychosocial Well-being and Satisfaction with Breasts of BREAST-Q and Body Image and Sexual Enjoyment of EORTC QLQ-BR23), but not on any of the Symptoms domains (Fig. 4 and Supplementary Table 4). The results of the univariable and adjusted multivariable regression analysis for cosmetic outcomes and HRQoL are shown in Table 3. Sexual Functioning and Sexual Enjoyment became statistically not significant in the adjusted analysis as well as Physical Functioning, whereas Social Functioning became statistically significant.

**Table 3:** Univariable and adjusted multivariable regression analysis with cosmetic outcome as independent variable and health-related quality of life as dependent variable. Adjusted for age, smoking and BMI (kg/m^2^).

	Unadjusted		Adjusted	
	β (95% CI)	p-value	β (95% CI)	p-value
Breast-Q				
Physical Well-being	−3.90 (−7.47 to −0.33)	**0.032**	−4.08 (−7.60 to −0.56)	**0.023**
Sexual Well-being	−8.31 (−12.52 to −4.11)	**<0.001**	−7.50 (−11.75 to −3.26)	**<0.001**
Psychosocial Well-being	−8.63 (−13.09 to −4.16)	**<0.001**	−7.27 (−11.76 to −2.79)	**0.001**
Satisfaction with Breasts	−13.13 (−16.15 to −10.10)	**<0.001**	−13.02 (−16.06 to −9.98)	**<0.001**
Side effects of Radiotherapy	−3.02 (−6.31 to 0.28)	0.072	−2.89 (−6.12 to 0.34)	0.079
EORTC QLQ-BR23				
Body Image	−5.68 (−8.81 to −2.54)	**<0.001**	−5.87 (−8.94 to −2.81)	**<0.001**
Sexual Functioning	−4.55 (−8.50 to −0.59)	**0.024**	−2.61 (−6.44 to 1.22)	0.181
Sexual Enjoyment	−6.36 (−11.96 to −0.77)	**0.026**	−3.79 (−9.22 to 1.64)	0.171
Future Perspectives	−4.09 (−8.65 to 0.48)	0.079	−4.46 (−8.95 to 0.04)	0.052
Breast Symptoms	2.74 (−0.23 to 5.72)	0.071	2.78 (−0.14 to 5.71)	0.062
Arm Symptoms	2.52 (−0.78 to 5.82)	0.134	2.33 (−0.88 to 5.54)	0.154
EORTC QLQ-C30				
Global Health status	−2.79 (−5.67 to 0.09)	0.058	−2.53 (−5.38 to 0.31)	0.081
Physical Functioning	−3.30 (−5.83 to −0.76)	**0.011**	−2.14 (−4.64 to 0.36)	0.093
Role Functioning	−3.04 (−6.42 to 0.33)	0.077	−2.97 (−6.37 to 0.44)	0.087
Emotional Functioning	−3.07 (−6.51 to 0.37)	0.080	−3.40 (−6.83 to 0.03)	0.052
Social Functioning	−3.24 (−6.55 to 0.07)	0.055	−3.35 (−6.65 to −0.04)	**0.047**
Fatigue	2.76 (−0.95 to 6.47)	0.144	2.52 (−1.16 to 6.21)	0.180
Pain	1.84 (−1.78 to 5.46)	0.319	1.42 (−2.15 to 5.00)	0.435
9-item cosmetic questionnaire	0.12 (0.07–0.17)	**<0.001**	0.12 (0.07–0.17)	**<0.001**

BREAST-Q Breast-Conserving Therapy module was used. EORTC-QLQ-BR23 = European Organization for Research and Treatment Quality of Life Questionnaire breast cancer module; EORTC-QLQ-C30 = European Organization for Research and Treatment Quality of Life Questionnaire Core 30; BMI = Body mass index; CI = Confidence Interval.

p-value statistically significant p <0.05 (stated in bold).

### 3.4 Association between severity of breast fibrosis and cosmetic outcomes

Patients with moderate/severe breast fibrosis were more likely to have a fair/poor cosmetic outcome (49 %) according to the BCCT.core software than patients with none/mild breast fibrosis (23 %) (OR 3.25, 95 %CI 2.21–4.79, p < 0.001) (Table 4A). The same association was observed for the patient-reported cosmetic outcome on the 9-item cosmetic questionnaire (OR 3.50, 95 %CI 2.07–5.91, p < 0.001) (Table 4B), although the agreement between the software and patient-reported cosmetic outcome was low (Cohen's kappa 0.074) (Table 5). The moderate/severe breast fibrosis group had significantly higher BRA, LBC and BOD median values compared to the none/mild breast fibrosis group (p < 0.001), indicating more asymmetry in the moderate/severe breast fibrosis group (Table 6).

**Table 4A:** BCCT.core cosmetic outcome by severity of breast fibrosis.

N = 749	None/mild breast fibrosis N = 595	Moderate/severe breast fibrosis N = 154	
BCCT.core cosmetic outcome:			
excellent/good	458 (77.0 %)	78 (50.6 %)	OR 3.25, 95% CI 2.21–4.79
fair/poor	137 (23.0 %)	76 (49.4 %)	p <0.001

Numbers shown as N (%); BCCT.core = Breast Cancer Conservation Treatment cosmetic results; OR = Odds Ratio; CI = Confidence Interval. p-value statistically significant p <0.05.

**Table 4B:** 9-item cosmetic questionnaire scores by severity of breast fibrosis.

N = 722	None/mild breast fibrosis N = 567	Moderate/severe breast fibrosis N = 155	
9-item cosmetic questionnaire			
≤1.5 = excellent/good	525 (92.6 %)	121 (78.1 %)	OR 3.50, 95% CI 2.07–5.91
>1.5 = fair/poor	42 (7.4 %)	34 (21.9 %)	p <0.001

Numbers shown as N (%); OR = Odds Ratio; CI = Confidence Interval.

p-value statistically significant p <0.05.

**Table 5:** Agreement between 9-item cosmetic questionnaire and BCCT.core cosmetic outcome.

N = 679	Excellent/good cosmetic outcome N = 500	Fair/poor cosmetic outcome N = 197	
9-item cosmetic questionnaire			
≤ 1.5 = excellent/good	465 (93.0 %)	160 (81.2 %)	Cohen's kappa 0.074,
>1.5 = fair/poor	35 (7.0 %)	37 (18.8 %)	95% CI 0.15–0.22

Numbers shown as N (%); CI = Confidence Interval.

**Table 6:** Association between the severity of breast fibrosis and cosmetic outcome evaluated by BCCT.core software using linear regression.

	None/mild breast fibrosis N = 595	Moderate/severe breast fibrosis N = 154	p-value
BRA	0.082 (0.052–0.113)	0.119 (0.074–0.175)	<0.001
LBC	0.040 (0.019–0.069)	0.076 (0.043–0.117)	<0.001
BOD	0.164 (0.120–0.216)	0.236 (0.165–0.302)	<0.001

Numbers shown as median relative values of BCCT.core software, with interquartile range between brackets. BCCT.core = Breast Cancer Conservation Treatment cosmetic results; BRA = Breast Retraction Assessment; LBC = Lower Breast Contour; BOD = Breast Overlap difference. Lower values of BRA, LBC and BOD represent less asymmetry.

p-value statistically significant p <0.05.

### 3.5 Comparison of the impact of moderate/severe breast fibrosis and unfavorable cosmetic outcomes on the different health-related quality of life domains

Both moderate/severe breast fibrosis and fair/poor cosmetic outcome were negatively associated with Sexual Well-being, Psychosocial Well-being, Satisfaction with Breasts and Body Image (Supplementary Tables 3 and 4). Furthermore, fair/poor cosmetic outcome was associated with significantly lower Sexual Functioning and Sexual Enjoyment scores, although not significant after adjusting for age, smoking and BMI. No significant association was found with the severity of breast fibrosis for these two domains. Additionally, it is important to note that patients with moderate/severe breast fibrosis reported more affected HRQoL domains compared to those with fair/poor cosmetic outcomes. Moreover, moderate/severe breast fibrosis was negatively associated with all Symptom domains, whereas no association between cosmetic outcomes and Symptom domains was found.

## 4 Discussion

To our knowledge, this is the first study that thoroughly assessed the impact of breast fibrosis and cosmetic outcomes on HRQoL in a large multicenter cohort of breast cancer patients who had been treated with BCT according to currently used oncoplastic surgery and radiotherapy techniques. We hypothesized that breast fibrosis negatively affects HRQoL and cosmetic outcomes and that unfavorable cosmetic outcomes have a negative impact on HRQoL as well. Our results suggest that both moderate/severe breast fibrosis and unfavorable cosmetic outcomes are associated with lower HRQoL scores. Important to note is that the Symptom domains of the questionnaires (i.e. Breast symptoms, Arm symptoms, Pain, and Fatigue) were negatively affected in the case of moderate/severe breast fibrosis, whereas this association was not found for unfavorable cosmetic outcomes. These outcomes were anticipated and seem reasonable, as it is understood that breast fibrosis can cause physical symptoms. The association between breast fibrosis and physical symptoms suggests that the impact of breast fibrosis extends beyond cosmetic concerns and can also lead to functional impairments, such as a restricted range of motion. These physical impairments can have a negative effect on patients' HRQoL by limiting their ability to perform daily activities.

Strikingly, our data furthermore suggest that moderate/severe breast fibrosis also has an impact on more HRQoL domains compared to unfavorable cosmetic outcomes. For example, moderate/severe breast fibrosis was statistically significant as well as clinically relevant associated to the domains Physical Well-being, Side effects of Radiotherapy, Future Perspectives, Global Health Status, Role Functioning, and Emotional Functioning, whereas unfavorable cosmetic outcomes were not. The finding that poor cosmetic outcomes negatively impact HRQoL is in line with other studies [[28], [29], [30]]. Hau et al. conducted a trial with 688 patients and found that poor cosmetic results were correlated with lower global HRQoL-C30 scores at 5 and 10 years post treatment [28]. Using patient self-evaluation and panel evaluation, Volders et al. found better scores for Body Image, Pain, Breast and Arm Symptoms in patients with excellent/good cosmetic outcomes compared to fair/poor cosmetic outcomes. However, no correlation was found between HRQoL and cosmetic outcomes when using BCCT.core [30]. The latter is partially in contrast with our results, as our study did show significantly better HRQoL scores regarding Body Image in patients with favorable cosmetic outcomes, evaluated by BCCT.core. In contrast to Volders et al. we found no correlation between unfavorable cosmetic outcomes and Symptom domains. Gao et al. found an association between breast fibrosis and Social Functioning assessed with EORTC QLQ-C30 [31], whereas this was statistically significant but not clinically relevant in our study.

In our cohort of 775 patients, 21 % had moderate/severe breast fibrosis, which seems in line with other studies, which have reported 10–30 % breast fibrosis after BCT [[8], [9], [10]]. We hypothesized that breast fibrosis results in unfavorable cosmetic outcomes, because fibrotic changes can distort breast shape and contour. On the other hand, it is crucial to recognize from our data that a significant number of patients with moderate/severe breast fibrosis can still achieve excellent cosmetic outcomes. This might be explained by the fact that for breast fibrosis scoring during physical examination, the maximum fibrosis score in the breast was used, meaning this could also only have been a small area in the breast. Still, we may conclude that breast fibrosis and cosmetic outcomes do not have a one-to-one association. This is supported by our findings that Symptom domains were only found statistically significant as well as clinically relevant for moderate/severe breast fibrosis and that breast fibrosis affects more HRQoL domains than unfavorable cosmetic outcomes. Efforts to achieve better cosmetic outcomes through surgical techniques or adjuvant therapies may inadvertently increase the risk of breast fibrosis if not carefully managed [32,33]. It highlights the need for future research aiming at minimizing the risk of developing fibrosis and maximizing good cosmetic outcomes to improve overall patient well-being after BCT.

Notably, our study had a high response rate to all questionnaires, specifically sexual domains. Moderate/severe breast fibrosis was negatively associated with the Sexual Well-being domain of BREAST-Q, but no association was found with the Sexual Functioning and Sexual Enjoyment domains of EORTC QLQ-BR23. This may appear contradictory, but a closer examination of the individual items of these questionnaires could provide an explanation. Whereas BREAST-Q focuses more on feeling attractive (with or without clothes) and confidence, EORTC QLQ-BR23 emphasizes whether someone is sexually active. Another explanation might be that patients do not like to fill out these questions concerning their sexuality and pretend that it is better than it actually is (Supplementary Table 2). However, it is important to pay attention to the Sexual domains, as it is often not discussed in the consultation room, yet an important aspect of HRQoL according to the World Health Organization [[34], [35], [36], [37], [38]].

### 4.1 Limitations

The aim of the analyses was to evaluate the effect of breast fibrosis on HRQoL (in several domains) and cosmetic outcomes, and the effect of cosmetic outcomes on HRQoL. Because patient characteristics such as age, BMI, and smoking may also have an effect on HRQoL, we adjusted the analyses for these characteristics. However, a limitation of this adjusted approach is that, if for example BMI has an effect on breast fibrosis, there may exist an indirect effect of BMI on HRQoL via the presence of breast fibrosis. Adjusting for BMI might thus have diluted the estimated effect of breast fibrosis on HRQoL found in the model. Therefore, we also fitted a model without these covariates as a sensitivity analysis. The adjusted and unadjusted approaches showed little differences, which indicates the robustness of our results. Another limitation of this study is that no baseline HRQoL data and breast photographs were available. This would have been the most ideal way to compare the results and the intra-patient reported outcomes. We also did not take the personality traits or feelings of depression of the patients into account, which might have influenced how the questionnaires were completed [11,39]. More patients declined participating in the study (49 %) than anticipated in advance. Various reasons were provided for this, including too much stress, having moved (too far) away, lack of travel allowance, a busy schedule, and religious beliefs preventing having a picture taken. These are not particularly reasons that potentially may have biased our results, however, this cannot completely be ruled out.  

In conclusion, the findings of this large multicenter study provide valuable insights into patient HRQoL after BCT, using contemporary oncoplastic surgical and radiotherapeutic techniques. Our study indicates that breast fibrosis and unfavorable cosmetic outcomes are negatively associated with various HRQoL domains. In addition, breast fibrosis is negatively associated with breast and arm symptoms, pain and fatigue, whereas unfavorable cosmetic outcomes are not. The present study corroborates the interrelated nature of breast fibrosis and cosmetic outcomes, although this is not a one-to-one association. These findings can be valuable for newly diagnosed breast cancer patients who are candidates for breast-conserving treatment, both in facilitating shared decision-making and in managing expectations. Future research should continue to explore which patient, tumor, systemic therapy, surgical and radiotherapy-related risk factors contribute to development of breast fibrosis and unfavorable cosmetic outcomes and the interplay between both oncoplastic surgery and radiotherapy techniques as part of BCT, ultimately improving the long-term HRQoL of breast cancer patients. This is currently ongoing in the STARLINGS study (ID NCT05263362).

## CRediT authorship contribution statement

**M.C.A.W. Notenboom:** Writing – original draft, Methodology, Investigation, Formal analysis, Data curation. **T.M.A.L. Klem:** Writing – review & editing, Conceptualization. **C.M.E. Contant:** Writing – review & editing, Conceptualization. **S.P. Ribbe:** Writing – review & editing, Investigation. **M. Franckena:** Writing – review & editing, Conceptualization. **J.J. Penninkhof:** Writing – review & editing, Conceptualization. **L.B. Koppert:** Writing – review & editing, Conceptualization. **P.W. Plaisier:** Writing – review & editing, Conceptualization. **M.A.M. Mureau:** Writing – review & editing, Conceptualization. **E.D. van Werkhoven:** Writing – review & editing, Methodology, Formal analysis, Conceptualization. **F.J.C. van der Veen:** Writing – review & editing, Conceptualization. **M. de Kraker:** Writing – review & editing, Conceptualization. **R.A. Nout:** Writing – review & editing, Supervision, Methodology, Conceptualization. **M.B.E. Menke-Pluijmers:** Writing – review & editing, Supervision, Methodology, Funding acquisition, Conceptualization. **F.E. Froklage:** Writing – review & editing, Supervision, Methodology, Funding acquisition, Conceptualization.

## Data availability

The data underlying this article are not available for public access at this time, as they include patient data.

## Ethical approval

This study was conducted in accordance with the ethical standards of the medical ethical committees of all participating hospitals (MEC-2021-0829), and in adherence to the legal requirements.

## Funding

M.C.A.W.N. received a salary from the BeterKeten foundation (Dutch: Stichting BeterKeten) and a research fund from the Albert Schweitzer Hospital for salary as well. The funders were not involved in the study design, data collection, data analysis and interpretation, manuscript preparation or publication decisions.

## Declaration of competing interest

The Department of Radiotherapy, Erasmus MC Cancer Institute has a research collaboration with Elekta AB (Stockholm, Sweden), Accuray Inc., (Sunnyvale, CA, USA) and Varian (Palo Alto, CA, USA). The current research was not funded by any of these companies. R.A.N. reports an additional research collaboration with the Dutch Cancer Society and Dutch Research Council. M.C.A.W.N. received a salary from the BeterKeten foundation (Dutch: Stichting BeterKeten) and a research fund from the Albert Schweitzer Hospital for salary as well. The funders were not involved in the study design, data collection, data analysis and interpretation, manuscript preparation or publication decisions. All other authors declared no conflict of interest.
