# Supplementary material for: The association between breast fibrosis, cosmetic outcomes, and long-term health-related quality of life after breast-conserving therapy: a multicenter cross-sectional observational cohort study
Source: Breast. 2025 Jul 14;83:104541. doi: 10.1016/j.breast.2025.104541 (PMC12355592; doi:10.1016/j.breast.2025.104541)
Supplement: Multimedia component 1 [file mmc1.docx]

**Supplementary Table 1:** Hypofractionation schedules without boost and with corresponding boost schedules

| Without boost | With boost (SIB)* | With boost (sequential) |
| --- | --- | --- |
| 40.05 Gy in 15 fr | 43.60 / 53.40 Gy in 20 fr | 40.05 Gy in 15 fr + boost 13.35 Gy in 5 fr (boost total 53.40 Gy) |
|  | 44.66 / 58.74 Gy in 22 fr |  |
| 42.56 Gy in 16 fr | 45.57 / 55.86 Gy in 21 fr | 42.56 Gy in 16 fr + boost 13.30 Gy in 5 fr (boost total 55.86 Gy) |
|  | 46.69 / 61.18 Gy in 23 fr |  |

Gy = gray; fr = fractions; SIB = simultaneously integrated boost

* whole breast dose / boost dose

**Supplementary Table 2:** Number of incomplete (domains of) questionnaires

| Questionnaires | Incomplete (N) |
| --- | --- |
| BREAST-Q  Physical Well-being  Sexual Well-being  Psychosocial Well-being  Satisfaction with Breasts  Side effects of Radiotherapy | 0  64  0  3  1 |
| EORTC QLQ-BR23 | 0 |
| EORTC QLQ-C30 | 0 |
| 9-item cosmetic questionnaire | 9 |

BREAST-Q Breast-Conserving Therapy module was used; EORTC-QLQ-BR23 = European Organization for Research and Treatment Quality of Life Questionnaire breast cancer module; EORTC-QLQ-C30 = European Organization for Research and Treatment Quality of Life Questionnaire Core 30.

**Supplementary Table 3:** Differences in health-related quality of life scores comparing patients with none/mild breast fibrosis and moderate/severe breast fibrosis

|  | Fibrosis N = 775 | | | | | |
| --- | --- | --- | --- | --- | --- | --- |
| HRQoL Questionnaires | None/mild  breast fibrosis  N = 616 (79%) | | Moderate/severe  breast fibrosis  N = 159 (21%) | | Δ  mean rash scores | p-value |
|  | Mean (SD) | Median (IQR) | Mean (SD) | Median (IQR) |  |  |
| BREAST-Q    Physical Well-being (N = 731)  Sexual Well-being (N = 667)  Psychosocial Well-being (N = 731)  Satisfaction with Breasts (N = 728)  Side effects of Radiotherapy (N = 730) | N = 573    74.5 (20.6)  66.4 (25.1)  74.8 (27.5)  75.5 (18.7)  86.7 (19.0) | 76.0 (66.0-89.0)  66.0 (50.0-91.0)  83.0 (62.0-100.0)  75.0 (61.0-100.0)  100.0 (78.0-100.0) | N = 158    62.1 (23.8)  60.6 (22.3)  65.9 (27.6)  63.6 (19.3)  76.6 (22.3) | 66.0 (45.0-76.0)  62.0 (48.0-73.0)  71.0 (55.0-84.0)  63.0 (51.0-75.0)  78.0 (65.0-100.0) | **12.4**  **5.8**  **8.9**  **11.9**  **10.1** | **<0.001**  **0.010**  **<0.001**  **<0.001**  **<0.001** |
| EORTC QLQ-BR23    Body Image  Sexual Functioning  Sexual Enjoyment  Future Perspectives  Breast Symptoms  Arm Symptoms | N = 569    89.7 (18.2)  26.3 (24.3)  34.2 (34.3)  71.9 (26.9)  12.8 (16.9)  11.2 (19.4) | 100.0 (83.3-100.0)  33.3 (0.0-33.3)  33.3 (0.0-66.7)  66.7 (66.7-100.0)  8.3 (0.0-16.7)  0.0 (0.0-11.1) | N = 154    83.4 (22.7)  26.7 (22.9)  34.4 (32.7)  63.2 (29.8)  21.9 (20.5)  16.3 (21.5) | 91.7 (75.0-100.0)  33.3 (0.0-33.3)  33.3 (0.0-66.7)  66.7 (58.3-75.0)  16.7 (8.3-33.3)  11.1 (0.0-22.2) | **6.3**  0.4  0.2  **8.7**  **–9.1**  **–5.1** | **<0.001**  0.889  0.887  **0.001** **<0.001**  **<0.001** |
| EORTC QLQ-C30    Global Health status  Physical Functioning  Role Functioning  Emotional Functioning  Social Functioning  Fatigue  Pain | N = 571    83.1 (16.9)  88.8 (15.1)  89.3 (20.1)  85.0 (20.2)  89.9 (19.5)  20.4 (22.1)  13.0 (21.0) | 83.3 (75.0-100.0)  93.3 (86.7-100.0)  100.0 (83.3-100.0)  91.7 (75.0-100.0)  100.0 (83.3-100.0)  22.2 (0.0-33.3)  0.0 (0.0-16.7) | N = 156    77.5 (18.8)  83.9 (16.8)  83.3 (22.1)  77.5 (23.0)  85.5 (22.3)  27.1 (24.4)  20.6 (25.1) | 83.3 (66.7-91.7)  86.7 (73.3-100.0)  100.0 (66.7-100.0)  83.3 (66.7-100.0)  100.0 (66.7-100.0)  22.2 (0.0-44.4)  16.7 (0.0-33.3) | **5.6**  4.9  **6.0**  **7.5**  4.4  **–6.7**  **–7.6** | **<0.001** <0.001 **<0.001** **<0.001**  0.013  **0.001** **<0.001** |

BREAST-Q Breast-Conserving Therapy module was used. HRQoL = Health-related Quality of life; SD = Standard deviation; IQR = Interquartile range; EORTC-QLQ-BR23 = European Organization for Research and Treatment Quality of Life Questionnaire Breast Cancer Module; EORTC-QLQ-C30 = European Organization for Research and Treatment Quality of Life Questionnaire Core 30.
Numbers in rash scores ranging from 0 to 100. Higher scores represent better outcomes or less side effects. Lower scores for Breast and Arm Symptoms, Fatigue and Pain represent better outcomes.
Δ mean rash scores ≥ 5 are considered clinically relevant.
p-value statistically significant p < 0.05.
p-values of statistically significant and clinically relevant domains are stated in bold.

**Supplementary Table 4:** Differences in health-related quality of life scores comparing patients with excellent/good cosmetic outcome and fair/poor cosmetic outcome

|  | Cosmetic outcome N = 749 | | | | | |
| --- | --- | --- | --- | --- | --- | --- |
| HRQoL Questionnaires | Excellent/good  cosmetic outcome    N=536 (72%) | | Fair/poor  cosmetic outcome  N=213 (28%) | | Δ  mean rash scores | p-value |
|  | Mean (SD) | Median (IQR) | Mean (SD) | Median (IQR) |  |  |
| BREAST-Q    Physical Well-being (N = 706)  Sexual Well-being (N = 646)  Psychosocial Well-being (N = 706)  Satisfaction with Breasts (N = 703)  Side effects of Radiotherapy (N = 705) | N = 505    73.0 (21.0)  67.6 (24.4)  75.7 (26.8)  76.9 (19.0)  85.5 (19.3) | 76.0 (63.0-89.0)  66.0 (53.0-91.0)  83.0 (64.0-100.0)  78.0 (62.5-100.0)  100.0 (78.0-100.0) | N = 201    69.1 (23.7)  59.3 (24.3)  67.0 (28.4)  63.7 (17.0)  82.4 (21.9) | 71.0 (52.0-89.0)  59.0 (43.8-73.0)  69.0 (54.0-87.0)  63.0 (51.0-75.0)  87.0 (71.0-100.0) | 3.9  **8.3**  **8.7**  **13.2**  3.1 | 0.084 **<0.001** **<0.001**  **<0.001**  0.132 |
| EORTC QLQ-BR23    Body Image  Sexual Functioning  Sexual Enjoyment  Future Perspectives  Breast Symptoms  Arm Symptoms | N = 501    90.2 (17.5)  27.8 (24.6)  36.3 (34.3)  71.3 (26.9)  13.8 (17.5)  11.5 (19.7) | 100.0 (83.3-100.0)  33.3 (0.0-33.3)  33.3 (0.0-66.7)  66.7 (66.7-100.0)  8.3 (0.0-16.7)  0.0 (0.0-11.1) | N = 198    84.5 (22.4)  23.2 (22.5)  30.0 (32.9)  67.2 (29.6)  16.6 (19.4)  14.0 (20.6) | 91.7 (75.0-100.0)  16.7 (0.0-33.3)  33.0 (0.0-66.7)  66.7 (66.7-100.0)  8.3 (0.0-25.0)  0.0 (0.0-22.2) | **5.7**  4.6  **6.3**  4.1  –2.8  –2.5 | **<0.001**  0.016  **0.025**  0.134  0.106  0.080 |
| EORTC QLQ-C30    Global Health status  Physical Functioning  Role Functioning  Emotional Functioning  Social Functioning  Fatigue  Pain | N = 502    82.7 (17.6)  88.8 (15.4)  89.0 (20.3)  84.4 (20.8)  89.9 (19.7)  20.8 (22.4)  13.9 (21.8) | 83.3 (75.0-100.0)  93.3 (85.0-100.0)  100.0 (83.3-100.0)  91.7 (75.0-100.0)  100.0 (83.3-100.0)  22.2 (0.0-33.3)  0.0 (0.0-16.7) | N = 200    79.9 (17.3)  85.5 (15.6)  86.0 (21.2)  81.3 (21.3)  86.7 (21.2)  23.6 (23.1)  15.7 (22.7) | 83.3 (66.7-91.7)  86.7 (73.3-100.0)  100.0 (66.7-100.0)  91.7 (66.7-100.0)  100.0 (66.7-100.0)  22.2 (0.0-33.3)  0.0 (0.0-33.3) | 2.8  3.3  3.0  3.1  3.2  –2.8  –1.8 | 0.024  0.001  0.043  0.038  0.045  0.146  0.355 |

BREAST-Q Breast-Conserving Therapy module was used. HRQoL = Health-related Quality of life; SD = Standard deviation; IQR = Interquartile range; EORTC-QLQ-BR23 = European Organization for Research and Treatment Quality of Life Questionnaire breast cancer module; EORTC-QLQ-C30 = European Organization for Research and Treatment Quality of Life Questionnaire Core 30.
Numbers in rash scores ranging from 0 to 100. Higher scores represent better outcomes or less side effects. Lower scores for Breast and Arm Symptoms, Fatigue and Pain represent better outcomes.
Δ mean rash scores ≥ 5 are considered clinically relevant.
p-value statistically significant p < 0.05.
p-values of statistically significant and clinically relevant domains are stated in bold.
